# Elucidation of Melanogenesis-Associated Signaling Pathways Regulated by Argan Press Cake in B16 Melanoma Cells

**DOI:** 10.3390/nu13082697

**Published:** 2021-08-04

**Authors:** Thouria Bourhim, Myra O. Villareal, Chemseddoha Gadhi, Hiroko Isoda

**Affiliations:** 1 Faculty of Sciences Semlalia, Cadi Ayyad University, Avenue Prince Moulay Abdellah, B.P. 2390, Marrakesh 40000, Morocco; t.bourhim@gmail.com; 2 Alliance for Research on the Mediterranean and North Africa (ARENA), University of Tsukuba, Tennodai 1-1-1, Tsukuba 305-8572, Japan; villareal.myra.o.gn@u.tsukuba.ac.jp; 3 Faculty of Life and Environmental Sciences, University of Tsukuba, Tennodai 1-1-1, Tsukuba 305-8572, Japan

**Keywords:** argan press-cake, MITF, JNK, cAMP/PKA, Wnt/β-catenin, microarray analysis

## Abstract

The beneficial effect on health of argan oil is recognized worldwide. We have previously reported that the cake that remains after argan oil extraction (argan press-cake or APC) inhibits melanogenesis in B16 melanoma cells in a time-dependent manner without cytotoxicity. In this study, the global gene expression profile of B16 melanoma cells treated with APC extract was determined in order to gain an understanding of the possible mechanisms of action of APC. The results suggest that APC extract inhibits melanin biosynthesis by down-regulating microphthalmia-associated transcription factor (*Mitf*) and its downstream signaling pathway through JNK signaling activation, and the inhibition of Wnt/β-catenin and cAMP/PKA signaling pathways. APC extract also prevented the transport of melanosomes by down-regulating *Rab27a* expression. These results suggest that APC may be an important natural skin whitening product and pharmacological agent used for clinical treatment of pigmentary disorders.

## 1. Introduction

*Argania spinosa* L. (family Sapotaceae) is a Moroccan endemic tree. It covers more than 800,000 hectares in the south-western region of the country [1]. In 1998, it was recognized as a biosphere reserve by the UNESCO and is known worldwide for its edible oil, which is now one of the world’s priciest oils [2]. Argan oil is extremely rich in unsaturated fatty acids and bioactive phytochemicals including tocopherols, phenolic compounds, and carotenoids [3] contributing to its pharmacological and cosmetic properties [4]. Traditionally, this oil is used as an anti-aging skin care [5]. Consumption of argan oil and/or topical application decrease trans-epidermal water loss and improve skin elasticity [6,7].

At agronomic level, this species has led to great financial returns, significantly reducing the poverty of the local population. In addition to its considerable socio-economic importance, argan trees also contribute to preventing soil erosion and desertification in the Southern part of Morocco [1]. However, destructive logging, climate change, and overexploitation have negatively affected the argan tree ecosystem and about 44% of the argan forest was lost between 1970 and 2007 [8]. The increase in demand for argan oil now requires a more efficient use of this valuable resource.

One of the by-products of argan oil production is argan press-cake (APC). While it was used previously as an animal feed, the discovery that it is abundant in functional secondary metabolites that give argan oil its functional properties made it a possible source of health benefits that argan oil users are looking for. APC contains a high phenolic compounds content that includes procyanidin B1 and B2, catechin, epicatechin, epigallocatechin gallate, phloridzin, myricetin, and quercitrin [9]. It also has a large amount of saponins, steroids, and triterpenoids [10], suggesting that APC could have the same biological effects as argan oil.

Skin exposure to ultraviolet (UV) radiation generates reactive oxygen species (ROS) that may cause skin aging and increased pigmentation [11]. Skin pigmentation is a result of melanin synthesis in the melanocytes, and its subsequent distribution to keratinocytes [12]. Melanin protects the skin against harmful UV radiation and stress resulting from exposure to various environmental pollutants [13]. However, increased production of melanin could cause several unwanted localized skin hyperpigmentation issues such as freckles and age spots, and these could be due to melasma and other post-inflammatory-associated hyperpigmentation [14]. The unwanted side effects of existing treatments for hyperpigmentation increase the demand for safe melanogenesis regulators of plant origin to treat skin hyperpigmentation diseases.

Melanin biosynthesis is regulated by a variety of signal transduction pathways that include cyclic adenosine monophosphate (cAMP) and mitogen activated protein kinase (MAPK), as well as the Wnt signaling pathway [15,16]. cAMP, via PKA, induces cAMP- response element-binding protein (CREB) family phosphorylation and activation, which then leads to the expression of microphthalmia transcription factor (MITF) [17], with CREB being one of the transcription factors that regulate MITF expression [18]. The transcription factor MITF is responsible for the expression of tyrosinase (TYR), tyrosinase related protein (TRP1), and dopachrome tautomerase (DCT), the major enzymes that catalyze relevant reaction in the melanogenesis process [17]. The Wnt pathway regulates MITF expression through the ß-catenin, the pivotal component of the Wnt pathway. When Wnt proteins bind to their receptors, it stabilizes the cytoplasmic ß-catenin, which then leads to its localization in the nucleus, where it regulates the expression of MITF [19]. The effect of the Wnt signaling pathway on MITF makes it an important pathway for regulating melanocyte differentiation [20].

Extracellular signal-regulated protein kinase (ERK), p38, and c-jun N-terminal kinase (JNK) have essential roles in melanogenesis regulation [21,22,23]. Interfering with p38, MAPK expression has been reported to promote melanogenesis and tyrosinase expression [24], while the active form of ERK, on the other hand, phosphorylates MITF at serine-73 during the posttranslational process, leading to its ubiquitination and, subsequently, its degradation [25]. JNK can interfere with CREB-regulated transcription co-activator 3 (CRTC3)-dependent MITF expression leading to melanogenesis inhibition [26].

Another key factor in melanogenesis regulation is the melanosome pH, which controls the maturation of melanosome in melanocytes and the rate of melanogenesis, as well as the ratio of eumelanin to phaeomelanin [27].

We have demonstrated the melanogenesis inhibitory effect of APC extract via *Mitf* expression down-regulation in B16 murine melanoma cells [28]. However, the mechanism by which APC regulates melanogenesis is not yet understood. In this study, the global gene expression analysis was done to identify the signaling involved in the melanogenesis inhibitory effect of APC extract.

## 2. Materials and Methods

### 2.1. Chemicals and Reagents

The Dulbecco’s modified eagle’s medium (DMEM), fetal bovine serum (FBS), and L-glutamine were from Sigma-Aldrich (Burlington, MA, USA). Penicillin/streptomycin solution was from Lonza, Walkersville Inc., (Walkersville, MD, USA). All the other chemicals were from Wako (Saitama, Japan).

### 2.2. Extraction of APC

Mature *Argania spinosa* fruits of the spherical type were harvested in July 2012 from the Sidi Ifni region (southwest of Morocco). Voucher samples (MARK10888-1) kept at the regional herbarium of Marrakech (Marrakesh, Morocco) were verified by Prof. Ahmed Ouhammou. The samples were dried at 25 °C, after which the argan fruits were manually peeled, and the nut’s shells cracked. The press-cake was obtained by extracting the oil from argan kernels using the mechanical press Komet DD 85 G press (IBG Monforts Oekotec GmbH & Co. KG, Mönchengladbach, Germany).

APC (10 g) was extracted with 100 mL ethanol 70% for 2 weeks at room temperature, after which it was centrifuged (1000× *g*, 15 min). The supernatant was then filter-sterilized using a 0.45 µm pore size filter (Millipore, Billerica, MA, USA) and kept at −80 °C in a freezer until use.

### 2.3. Cells and Cell Culture

The B16 murine melanoma cells used in this study were purchased from Riken Cell Bank (Tsukuba, Japan). Cells were cultured in DMEM supplemented with 10% FBS, 4 mmol/L L-glutamine, 50 units ⁄ml penicillin, and 50 µg/mL streptomycin, and they were maintained in a humidified incubator at 37 °C with 5% CO_2_.

### 2.4. Total RNA Extraction

B16 cells were seeded at a density of 3 × 10^6^ cells per 100-mm Petri-dish and were allowed to attach before treatment. The growth medium was replaced with a fresh one containing 0, 50 µg/mL of APC extract or 100 µmol/L of arbutin. The extraction of total RNA was done as described previously [28]. After the specified treatment time, the cells were washed twice with cold PBS before RNA extraction using the ISOGEN kit (Nippon Gene, Tokyo, Japan). The quality and quantity of the RNA was examined using a Nanodrop 2000 spectrophotometer (Nanodrop Technologies, Wilmington, DE, USA). The RNAs used were reverse transcribed using the SuperScript III Reverse Transcriptase Kit (Invitrogen, Carlsbad, CA, USA).

### 2.5. Quantitative Real-Time PCR Analysis

The effect of APC on gene expression in B16 cells was determined by real-time PCR (Applied Biosystems, Carlsbad, CA, USA) using TaqMan master mix, and performed using the TaqMan 7500 Fast Real-time PCR System (Applied Biosystems, Carlsbad, CA, USA). Cycling conditions were as follows: 2 min at 50 °C and 10 min at 95 °C, followed by 40 cycles of 95 °C for 15 s and 60 °C for 1 min. The assay IDs of the TaqMan primers used were: *Rab27a*—Mm00469997_m1, *Ctnnb1*—Mm00483039_m1, *Map3k12*—Mm00437378_m1, and *Gapdh*–Mm99999915_g1. *Gapdh* was used as the internal control. All the reactions were run in triplicates.

### 2.6. DNA Microarrays

The microarray analysis was performed to determine the global transcriptional response of B16 melanoma cells to argan press-cake or arbutin (positive control) treatment. Total RNA (100 ng) was reverse transcribed to synthesize the first-strand cDNA, which was then converted into double-stranded cDNA (ds-cDNA). The ds-cDNA template and biotin-labeled aRNA was generated using the 30 IVT Express Labeling Kit (Affymetrix, Santa Clara, CA, USA). The ds-cDNA was used to synthesize biotin-modified aRNA, 10 μg of which was fragmented using the GeneAtlas 3 IVT Express Kit, before hybridization to the Affymetrix Mouse 430 PM Array strips (Affymetrix) for 16 h at 45 °C. Following hybridization, the microarray DNA array was washed and stained in the GeneAtlas Fluidics Station 400 (Affymetrix), and then scanned using the GeneAtlas Imaging Station (Affymetrix). Data analysis was carried out using Affymetrix Expression Console Software and Affymetrix Transcriptome Analysis Console (TAC) 2.0 Software (Affymetrix). Hierarchical clustering was performed using Euclidean distance by TIGR’s MultiExperiment Viewer v4.9.0 software. The rows represent genes while columns represent the experimental samples. The heat map represents the gene expression ratios with the green and red color of cells indicating gene down- and up-regulation. In addition, the DNA microarray data have been deposited in the ArrayExpress database at EMBI-EBI (Available online: www.ebi.ac.uk/arrayexpress (accessed on 27 May 2020)), under the reference number E-MTAB-9089.

### 2.7. Statistical Analysis

The results were expressed as mean ± standard deviation (SD) of three independent experiments. The differences between means were analyzed for significance using one-way analysis of variance (ANOVA) with a Fisher’s Least Significant Difference (LSD) post hoc test. A value of *p* ≤ 0.05 was considered significant.

## 3. Results

### 3.1. Gene Expression Profile of APC Extract-Treated Cells

The expression level of genes in APC extract- or arbutin-treated cells was determined. Furthermore, genes expressed in APC-treated cells that were 1.5-fold different from the untreated controls were subjected to GO Enrichment Analysis. GO Enrichment Analysis identifies the genes that are relevant to biological processes and that were affected by APC treatment. The genes that were significantly changed in expression are presented in Table 1. The processes down-regulated by APC extract included those that are associated with pigmentation, melanosome transport, keratinocyte differentiation, melanocyte differentiation, cell differentiation, and apoptosis. Moreover, nervous system development, Wnt receptor signaling pathway through beta-catenin, the glutathione metabolic process, and MAP kinase kinase kinase activity were differentially regulated by APC extract.

DNA microarray analysis also revealed that several Wnt signaling pathway associated genes were significantly changed in response to APC extract treatment (Table 1). Among those genes, several genes were down-regulated (*Ctnnb1* and *Cxxc4*), while some were up-regulated (*Tcf7L2*). Interestingly, a number of intermediate genes of the MAPK pathway were up-regulated (*Akap9*, *Rasa1*, *Spry4*), while *Map3k12* was down-regulated by APC extract treatment. Moreover, the microarray dataset revealed an up-regulation by the APC extract of the *Prkar1b* gene, a regulatory subunit of PKA inactivating its catalytic domains. Additionally, several genes under the transcriptional regulation of MITF, such as *Tyr*, *Trpm1*, *Vat1*, *Atp6v0b*, *Rbm39*, and *Usp9x*, were significantly down-regulated, including *Rab27a*, which is a melanosome transport protein. The obtained data also showed that many of the solute carrier genes (SLCs) (*Slc24a4*, *Slc35a5*, *Slc12a6*, *Slc6a6*, *Slc4a4*, *and Slc7a11*) were differentially expressed following APC extract treatment (Table 1).

**Figure 1:**
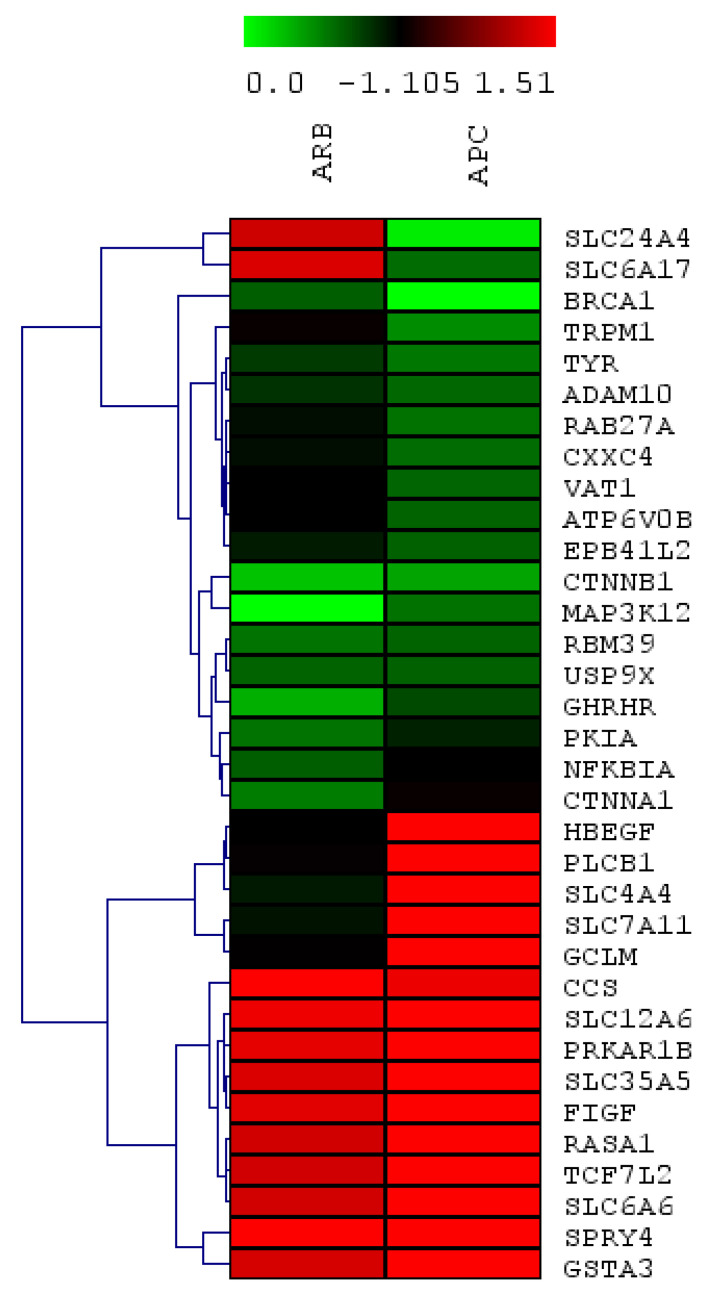
Heat map and hierarchical clustering of 34 genes in response to treatment with argan press-cake (APC) or by arbutin (ARB) in B16 melanoma cells. Clustering was calculated using Euclidian distance in the TIGR’s MultiExperiment Viewer v4.9.0 software. Rows and columns represent genes and experimental samples, respectively. Gene expression ratios are presented in the heat map with green and red color indicating down and up-regulation, respectively.

Genes that were significantly modulated by APC extract or arbutin were subjected to hierarchical clustering and the results grouped the genes into two main groups: APC-down-regulated and APC-up-regulated genes (Figure 1). The down-regulated genes formed five subgroups, while up-regulated genes formed three subgroups. The first cluster is composed of genes relevant to transporter activity represented by two solute carrier genes *Slc24a4* and *Slc6a17* (*p* = 0.52). The second cluster are genes significant for DNA repair (*Brca1*, *p* = 1.04). The next cluster constituted of genes that are relevant in melanin synthesis, melanosome transport, ATP binding, ion transport, and metal binding (*Tyr*, *Rab27a*, *Trpm1*, *Vat1*, *Atp6v0b*, *Cxxc4*, *Adam10*, *Epb41l2* (*p* = 0.30)). In addition, a regulation of *Ctnnb1*, *Map3k12* genes that play a role in the Wnt signaling pathway and MAP kinase activity was observed in cluster four (*p* = 0.34). The fifth cluster are genes in the Wnt signaling pathway and c-AMP signaling pathway, or those that function in metal binding, protein binding (*p* = 0.33). The last three clusters represent the up-regulated genes by APC extract treatment. Those genes play roles in the c-AMP signaling pathway, Wnt signaling pathway, transporter activity, protein binding, and kinase activity (Figure 1, Table 2).

### 3.2. Validation of Global Gene Expression Results

**Figure 2:**
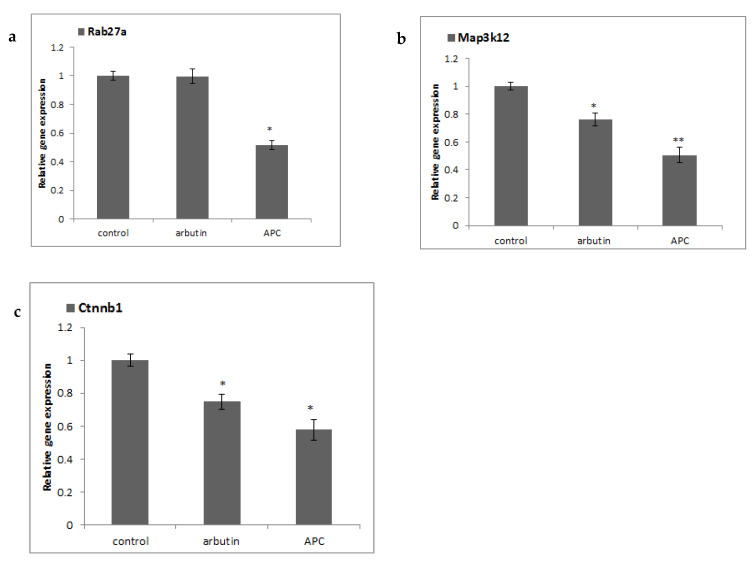
Effect of argan press-cake (APC) on the expression of (**a**) *Rab27a* (member RAS oncogene family), (**b**) mitogen-activated protein kinase kinase kinase 12 (*Map3k12*), and (**c**) catenin (cadherin associated protein) beta1 (*Ctnnb1*) genes in B16 cells. Cells were seeded onto a 100-mm dish at a density of 3 × 10^6^. B16 cells were treated with or without arbutin (100 µM) or argan press-cake extract (50 µg/mL) for 24 h. Rt-PCR was used to determine the select genes expression level. Data are expressed as mean ± SD (*n* = 3), * *p* < 0.05, ** *p* < 0.01.

The DNA microarray results were validated using rt-PCR. *Rab27a* was significantly down-regulated by 51.7%. *Ctnnb1* (catenin cadherin associated protein beta1) and *Map3k12* (mitogen-activated protein kinase kinase kinase 12), the genes that code for proteins that regulate *Mitf* expression, were decreased significantly (Figure 2). The changes in genes expression were consistent with the microarray data.

## 4. Discussion

Previously, we demonstrated that APC extract has a melanogenesis inhibitory effect in B16 cells. APC inhibits the melanogenic enzymes expression through *Mitf* down-regulation [28]. In this study, the global gene expression in the B16 cells in response to APC treatment was analyzed in order to fully understand the effect of APC on B16 cells and to identify APC targets that may have directly or indirectly contributed to its inhibitory effect, specifically on MITF and on melanogenesis in general.

As presented in Table 1, APC up-regulated the *Prkar1b* gene, which codes for the protein that is a regulatory subunit of cyclic AMP-dependent protein kinase A (PKA) [29]. As previously reported, APC treatment inhibits the *Mitf* expression [28], which could be due to the inhibition of signals that are upstream of *Mitf*. In short, an inactivation of PKA and the down-regulation of cAMP due to APC treatment could explain the decreased *Mitf* expression. Down-regulation of *Mitf* also led to inhibition of melanosome transport by its direct regulatory effect on *Rab27a* expression [30]. In this study, *Rab27a* expression was down-regulated by 51.7%. In addition, it has also been observed that *β-catenin* was down-regulated. β-catenin, as a transcriptional regulator, can be redirected by MITF away from genes that are under the regulation of the Wnt signaling toward *Mitf*-specific targets [31]. Additionally, β-catenin is also known to regulate *Mitf* expression [32]. β-catenin may be degraded via a ubiquitin-dependent, PKA-attenuated GSK3β (glycogen synthase kinase-3β) action [24]. MITF-regulated gene *Rab27a*, in addition to its function in melanosome transport, also has a significant role in various cell activities that include cell growth, invasion, and metastasis [33,34]. Several studies have associated increased expression of *Rab27a* with carcinogenesis, and it has been reported to promote the stemness of colon cancer cells [35], leading to poor survival in pancreatic cancer [36]. The inhibition of *Rab27a* by APC therefore suggests an anti-cancer effect of APC by targeting *Rab27a.*

Cheli et al. (2009) showed that cAMP controls the melanosome pH through PKA-independent mechanism. It has also been suggested that cAMP modulates vacuolar ATPases and ion transporters expression [37]. The melanosome pH of light-colored human skin melanocytes is more acidic, which explains the observed low tyrosinase activity observed in Caucasian skin. In contrast, melanosomes in dark human skin are less acidic and have higher tyrosinase activity [38]. The results of this study revealed that many of the solute carrier genes (*Slc24a4*, *Slc35a5*, *Slc12a6*, *Slc6a6*, *Slc4a4*, *and Slc7a11*), several vacuolar ATPases, and ion transporters were differentially expressed following APC extract treatment. In addition to its action at the mRNA level, the results of this study further suggest that APC extract may decrease the activity of TYR by reducing the pH of melanosome. This may also explain why despite the fact that there was no change in the TYR expression, a decrease in B16 cells melanin content at 48 h was observed [28]. Moreover, a growing number of solute carrier genes have been reported to play pivotal roles in melanogenesis regulation and in ethnic skin color determination [39,40], and this includes *Slc24a4*, which was down-regulated by 2-fold in APC extract-treated cells.

**Figure 3:**
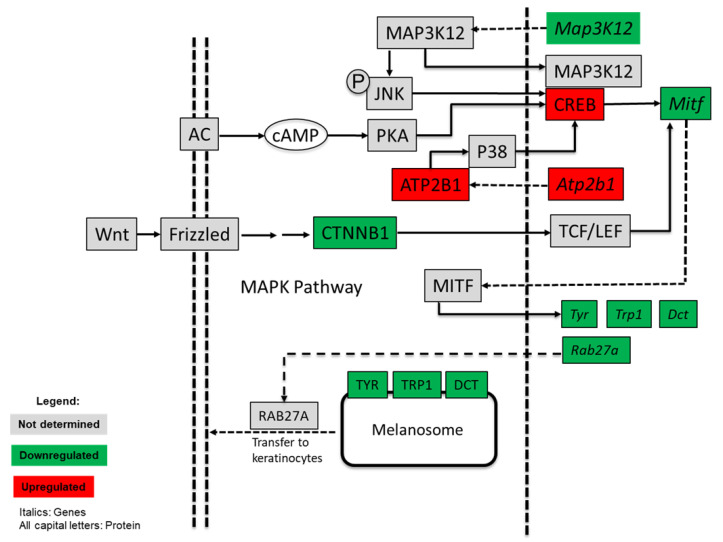
Argan press-cake (APC) down-regulated genes relevant to melanogenesis. This figure is adapted from Kyoto Encyclopedia of Genes and Genomes (KEGG) Pathway for melanogenesis (Available online: https://www.genome.jp/pathway/map04916 (accessed on 15 February 2021)).

The effect of APC extract on the JNK signaling most likely contributed to the decreased melanogenesis. JNK is regulated by several molecules, but in this study, a down-regulation of the expression of *Map3k12* and up-regulation of *Plcb1* could induce the activation of the JNK pathway (Figure 3). The activation of JNK causes phosphorylation of MITF at serine 73, which could then result to subsequent ubiquitin-dependent proteasomal degradation of MITF [21]. Although MITF was observed to be down-regulated at the transcriptional level, it is also possible that post-transcriptional modifications occurred in response to APC extract treatment. That effect could be through the several pathways mentioned earlier, which include the MAPK (JNK) pathway.

DNA microarray results also showed that the Wnt/β-catenin and the cAMP/PKA signaling pathways were inactivated (Figure 3). The cAMP’s effect on melanogenesis is mainly of its effect on the process’ rate limiting enzyme tyrosinase, and cAMP stimulating tyrosinase activity [41]. However, what has been well reported is its effect on PKA activation that will then lead to CREB activation. CREB activates MITF expression. Compounds that inhibit the cAMP pathway are expected to inhibit melanogenesis [42].

This complex effect of APC extract on B16 cells could be attributed to the presence of different bioactive compounds, polyphenols, saponins, steroids, and triterpenoids.

In this study, the APC-treated cells were not just compared to untreated cells, but also to arbutin, the positive control. Arbutin is a D-glucopyranoside derivative of hydroquinone, which is a widely used skin lightning agent in cosmetic and healthcare industry [43,44]. Our microarray data revealed that *Nfkbia*, which is involved in nuclear factor kappa B (NF-κB) binding, and cytoplasmic sequestering were down-regulated by arbutin. NF-κB signaling has been reported to play a key role in melanogenesis regulation [45,46,47]. In addition, Ahn et al. (2003) reported that the treatment of human keratinocytes with arbutin effectively down-regulate NF-κB activation, which is consistent with our results [48]. It is well-known that the MAP3K-related kinase is associated with NF-κB stimulation by TNF, CD95, and IL-1 [49,50]. Therefore, *Map3k12*, which was significantly decreased by arbutin, appears to be a plausible target in regulating melanogenesis through NF-κB inhibition.

## 5. Conclusions

Argan press-cake extract significantly decreases melanin synthesis in B16 cells [28]. In this study, microarray analysis sheds light on the exact mechanism by which the anti-melanogenesis effect occurs. The inhibitory effect on melanogenesis was a result of the regulation of several signals at once, including (i) the inactivation of the cAMP/PKA signaling pathway through an up-regulation of *Prkar1b* gene that lead to PKA down-regulation and subsequently inhibition of *Mitf* expression; (ii) the down-regulation of the Wnt/β-catenin signaling pathway; and (iii) the activation of the JNK MAP kinase pathway. JNK activation causes a subsequent ubiquitin-dependent proteasomal degradation of MITF. In addition, APC may inhibit not only the melanin production but also melanosome transport by *Rab27a* down-regulation.

By inhibiting the overproduction and accumulation of melanin in the skin that cause numerous related pigment disorders like melasma, freckles or lentigines, dermatitis, and geriatric skin pigmentation [14], APC may become an alternative natural and noncytotoxic therapeutic agent against hyperpigmentation disorders. It also has a potential use in cosmetics like argan oil [51] and other argan oil extraction by-products, argan fruit shells [52], argan leaves [53], etc.

## Figures and Tables

**Table 1:** List of genes that were differentially expressed (1.5-fold change in expression) in B16 melanoma cells treated with argan press-cake (APC) or arbutin (ARB) as determined by DNA microarray (*p* < 0.05) ^1^.

Gene Symbol	Gene Name	Function	Fold Change	
			ARB	APC
*Brca1*	Breast cancer 1	Double-strand break repair via homologous recombination, DNA repair, lipid metabolic process	−1.5	−2.5
*Slc24a4*	Solute carrier family 24 (sodium/potassium/calcium exchanger), member 4	Calcium, potassium: Sodium transporter activity	1.0	−2.1
*Ctnnb1*	Catenin (cadherin associated protein), beta 1	Wnt receptor signaling pathway, positive regulation of I-kappaB kinase/NF-kappaB cascade, positive regulation of MAPK cascade, skin development	−1.1	−1.8
*Trpm1*	Transient receptor potential cation channel, subfamily M, member 1	G-protein coupled glutamate receptor signaling pathway, calcium ion transport into cytosol	−1.0	−1.7
*Oca2*	Oculocutaneous albinism II	Transport, spermatid development, cell proliferation, melanocyte differentiation, melanin biosynthetic process, pigmentation, developmental pigmentation, transmembrane transport	−1.0	−1.7
*Tyr*	Tyrosinase	Melanin biosynthetic process	−1.0	−1.6
*Rab27a*	RAB27A, member RAS oncogene family	Protein transport, melanocyte differentiation, melanosome localization, melanosome transport, pigmentation	−1.2	−1.6
*Map3k12*	Mitogen-activated protein kinase kinase kinase 12	Activation of MAPKK activity, protein phosphorylation, JNK cascade	−2.3	−1.6
*Slc6a17*	Solute carrier family 6 (neurotransmitter transporter), member 17	Neurotransmitter: sodium symporter activity, neurotransmitter transport	1.2	−1.6
*Vat1*	Vesicle amine transport protein 1 homolog (T californica)	Zinc ion binding, oxidoreductase activity, negative regulation of mitochondrial fusion	−1.1	−1.6
*Atp6v0b*	Atpase, H+ transporting, lysosomal V0 subunit B	Hydrogen-exporting ATPase activity, phosphorylative mechanism, hydrogen ion transmembrane transporter activity ATP catabolic process, ion transport, ATP hydrolysis coupled proton transport	−1.1	−1.5
*Rbm39*	RNA binding motif protein 39	Nucleotide binding, transcription coactivator activity, poly(A) RNA binding, regulation of transcription, DNA-templated	−1.6	−1.5
*Usp9x*	Ubiquitin specific peptidase 9, X chromosome	Cysteine-type peptidase activity, hydrolase activity, transforming growth factor beta receptor signaling pathway, BMP signaling pathway, hippocampus development	−1.5	−1.5
*Ccs*	Copper chaperone for superoxide dismutase	ROS catabolism, superoxide dismutase copper chaperone activity	1.5	1.4
*Hbegf*	Heparin-binding EGF-like growth factor	Positive regulation of keratinocyte migration, positive regulation of protein kinase B signaling cascade, positive regulation of wound healing	−1.1	1.5
*Plcb1*	Phospholipase C, beta 1	Enzyme binding, positive regulation of JNK cascade,	−1.1	1.5
*Prkar1b*	Protein kinase, camp dependent regulatory, type I beta	Camp-dependent protein kinase inhibitor activity, camp-dependent protein kinase regulator activity, regulation of protein phosphorylation	1.3	1.5
*Slc7a11*	Solute carrier family 7 (cationic amino acid transporter, y+ system), member 11	Amino acid transmembrane transporter activity, response to toxic substance, platelet aggregation	1.1	1.5
*Maoa*	Monoamine oxidase A	Primary amine oxidase activity, oxidoreductase activity, dopamine catabolic process	−1.2	1.5
*Mcm3*	Minichromosome maintenance deficient 3	DNA replication initiation	1.5	1.6
*Erbb3*	V-erb-b2 erythroblastic leukemia viral oncogene homolog 3 (avian)	Protein tyrosine kinase activity, receptor signaling protein tyrosine kinase activity, protein phosphorylation	1.1	1.6
*Rasa1*	RAS p21 protein activator 1	Positive regulation of Ras GTPase activity, negative regulation of Ras protein signal transduction	1.1	1.6
*Slc4a4*	Solute carrier family 4 (anion exchanger), member 4	Transporter activity, inorganic anion exchanger activity, sodium/bicarbonate symporter activity, regulation of pH	−1.2	1.6
*Dzip3*	DAZ interacting protein 3, zinc finger	Zinc ion binding	−1.1	1.6
*Slc35a5*	Solute carrier family 35, member A5	Nucleotide-sugar transmembrane transporter activity, carbohydrate transport	1.2	1.6
*Slc12a6*	Solute carrier family 12, member 6	Potassium/chloride symporter activity, ion transport, cation chloride transport	1.4	1.6
*Cbl*	Casitas B-lineage lymphoma	Phosphotyrosine binding, positive regulation of phosphatidylinositol 3-kinase cascade, protein binding, calcium ion binding, zinc ion binding	1.2	1.6
*Tcf7l2*	Transcription factor 7 like 2, T cell specific, HMG box	Protein kinase binding, skin development, canonical Wnt receptor signaling pathway involved in positive regulation of epithelial to mesenchymal transition	1.0	1.6
*Figf*	C-fos induced growth factor	Protein binding, vascular endothelial growth factor receptor binding	1.2	1.6
*Ccrl1*	Atypical chemokine receptor 4	G-protein coupled receptor signaling pathway, scavenger receptor activity	1.5	1.7
*Slc6a6*	Solute carrier family 6 (neurotransmitter transporter, taurine), member 6	Neurotransmitter/sodium symporter activity, beta-alanine transport	1.1	1.7
*Slc4a4*	Solute carrier family 4 (anion exchanger), member 4	Anion transmembrane transporter activity, sodium/bicarbonate symporter activity, regulation of pH, bicarbonate transport	1.1	1.7
*Braf*	Braf transforming gene	MAP kinase kinase kinase activity, activation of MAPKK activity, positive regulation of ERK1 and ERK2 cascade	1.1	1.7
*Akap12*	A kinase (PRKA) anchor protein 13	Regulation of protein kinase activity, phosphorylation	1.2	1.7
*Akap13*	A kinase (PRKA) anchor protein (gravin) 12	Positive regulation of protein kinase A signaling cascade	1.1	1.7
*Tpr*	Translocated promoter region	MAPK import into nucleus	1.1	1.8
*Crebbp*	CREB binding protein	Negative regulation of transcription from RNA polymerase II promoter, p53 binding	−1.0	1.8
*Adh7*	Alcohol dehydrogenase 7 (class IV), mu or sigma polypeptide	Aldehyde oxidase activity, oxidoreductase activity	1.1	1.9
*Cxcl10*	Chemokine (C-X-C motif) ligand 10	Protein secretion	1.4	1.9
*Taok1*	TAO kinase 1	Protein kinase activator activity, protein phosphorylation	1.2	1.9
*Atp2b1*	Atpase, Ca++ transporting, plasma membrane 1	Nucleotide binding, hydrolase activity, calcium ion transport	1.1	2.2
*Slc7a11*	Solute carrier family 7 (cationic amino acid transporter, y+ system), member 11	Amino acid transmembrane transporter activity, response to toxic substance, platelet aggregation	−1.2	2.2
*Gclm*	Glutamate-cysteine ligase, modifier subunit	Glutamate-cysteine ligase activity, glutamate-cysteine ligase catalytic subunit, protein heterodimerization activity, cysteine, glutamate and glutathione metabolic process, response to oxidative stress, apoptotic mitochondrial changes, negative regulation of neuron apoptotic process, negative regulation of extrinsic apoptotic signaling pathway	−1.1	2.3
*Spry4*	Sprouty homolog 4 (Drosophila)	Protein binding, multicellular organismal development, regulation of signal transduction, negative regulation of MAP kinase activity	1.5	2.4
*Gsta3*	Glutathione S-transferase, alpha 3	Glutathione transferase activity, metabolic process	1.1	2.7

^1^ Based on gene ontology annotations in Mouse Genome Informatics (MGI).

**Table 2:** Gene clusters obtained by hierarchical clustering of significantly expressed genes in APC-treated B16 cells ^a,b^.

Cluster No. (*p*-Value)	Signaling Pathway	Genes
1 (0.52)	Transporter activity	*Slc24a4*, *Slc6a17*
2 (1.04)	DNA repair	*Brca1*
3 (0.30)	Melanogenesis regulation, melanosome transport, ATP binding, ion transport, metal binding	*Tyr*, *Rab27a*, *Trpm1*, *Vat1*, *Atp6v0b*, *Cxxc4*, *Adam10*, *Epb41l2*
4 (0.34)	Wnt signaling pathway, MAP kinase activity	*Ctnnb1*, *Map3k12*
5 (0.33)	Wnt signaling pathway, metal binding, protein binding c-AMP signaling pathway	*Rbm39*, *Usp9x*, *Ghrhr*, *Pkia*, *Nfkbia*, *Ctnna1*
6 (0.68)	Positive regulation of keratinocyte migration, positive regulation of JNK cascade, transporter activity, glutamate-cysteine ligase activity	*Hbegf*, *Plcb*, *Slc4a4*, *Slc7a11*, *Gclm*
7 (0.37)	Ros catabolism, transporter activity, cAMP dependent regulatory, protein binding, regulation of Ras GTPase activity, Wnt signaling	*Ccs*, *Slc12a6*, *Prkar1b*, *Slc35a5*, *Figf*, *Rasa1*, *Tcf7l2*, *Slc6a6*
8 (0.50)	Protein binding, MAP kinase activity, glutathione transferase activity	*Spry4*, *Gsta3*

^a^ Euclidean distance by TIGR’s MultiExperiment Viewer v4.9.0 software. ^b^ Based on gene ontology annotations in Mouse Genome Informatics (MGI).

## Data Availability

The DNA microarray data have been deposited in the ArrayExpress database at EMBI-EBI (Available online: www.ebi.ac.uk/arrayexpress (accessed on 27 May 2020)), under the reference number E-MTAB-9089.
